# Carney complex: a case with thyroid follicular adenoma without a PRKAR1A mutation

**DOI:** 10.1186/s40792-018-0438-z

**Published:** 2018-04-17

**Authors:** Shinji Hattori, Yukou Yamane, Ryoichi Shimomura, Yuki Uchida, Nobuhiko Toyota, Yoshio Miura, Setsujyo Shiota, Yoshitsugu Tajima

**Affiliations:** 1 Department of Surgery, Masuda Red Cross Hospital, 103-1 Otoyoshi, Masuda, Shimane 698-8501 Japan; 2 Yamane Hospital, 1517-1 Atsuta, Hamada, Shimane 697-0062 Japan; 3 Department of Pathology, Masuda Red Cross Hospital, 103-1 Otoyoshi, Masuda, Shimane 698-8501 Japan; 4 Department of Digestive and General Surgery, Faculty of Medicine, Shimane University, 89-1 Enya, Izumo, Shimane 693-8501 Japan

**Keywords:** Carney complex, Thyroid adenoma, Cardiac myxoma

## Abstract

**Background:**

Carney complex (CNC) is a very rare disease. Although thyroid lesions are included in the diagnostic criteria for CNC, they are an infrequent occurrence.

**Case presentation:**

The patient was a 69-year-old woman who had undergone the removal of a left atrial myxoma 10 years earlier, at the age of 59. At the time of the operation, thyroid ultrasonography (US) revealed multiple hypoechoic nodules. Thyroid scintigraphy revealed an increased uptake of ^99m^Tc in these lesions, which was consistent with toxic multinodular goiter, and she was diagnosed with CNC. Genetic studies showed no mutation in the PRKAR1A (protein kinase A regulatory subunit 1-α) gene. From then on, she received annual brain magnetic resonance imaging (MRI), abdominal computed tomography (CT), and thyroid US examinations. Her follicular thyroid nodules gradually increased in number and size. Although aspiration cytology samples from the thyroid nodules diagnosed them as class III, thyroid cancer could not be ruled out. The patient underwent a partial thyroidectomy, and the pathological diagnosis was multiple follicular adenomas.

**Conclusion:**

Careful and frequent evaluation of the thyroid gland should be required for CNC patients due to the potential for carcinoma to develop in the context of a variety of follicular thyroid lesions.

## Background

Carney complex (CNC) is a familial tumor syndrome first reported by J. Aidan Carney in 1985. The diagnostic criteria include myxoma, endocrine overactivity, and spotty skin pigmentation. CNC is a very rare disease, with about 750 cases worldwide and only 33 cases in Japan having been reported to date. Half of CNC is inherited in an autosomal dominant fashion, and the rest is sporadic. The causative genes of CNC are located at 2p16 (CNC type 2) or 17q2 (CNC type 1), and they are heterogeneous in this disorder [1]. PRKAR1A (protein kinase A regulatory subunit 1-α) was identified in 2000 as a causative gene of CNC type 1 but has not been identified in CNC type 2 [1].

In past reviews, 10% of CNC patients had carcinomatous thyroid lesions. The data also suggests that patients with CNC are more susceptible to the development of thyroid carcinoma than those without, and patients with a PRKAR1A mutation are at a higher risk of developing thyroid tumors [2]. In patients with CNC, therefore, careful and frequent evaluation of the thyroid gland is necessary.

## Case presentation

A 69-year-old Japanese female was admitted to our hospital due to thyroid tumors that had been increasing in number and size on routine thyroid ultrasonography (US). On physical examination, the patient was in a good nutritional state and in no acute distress. She had no symptoms of heart failure. There was a palpable soft mass in the thyroid gland, and the patient’s serum CEA level was a little high (5.1 ng/mL). Free T3 and free T4 levels were normal, but her TSH level was high (4.56 μIU/mL). Full blood count, blood chemistry, and serum thyroglobulin levels were normal. She had been taking thiamazole for 25 years for hyperthyroidism.

**Fig. 1:**
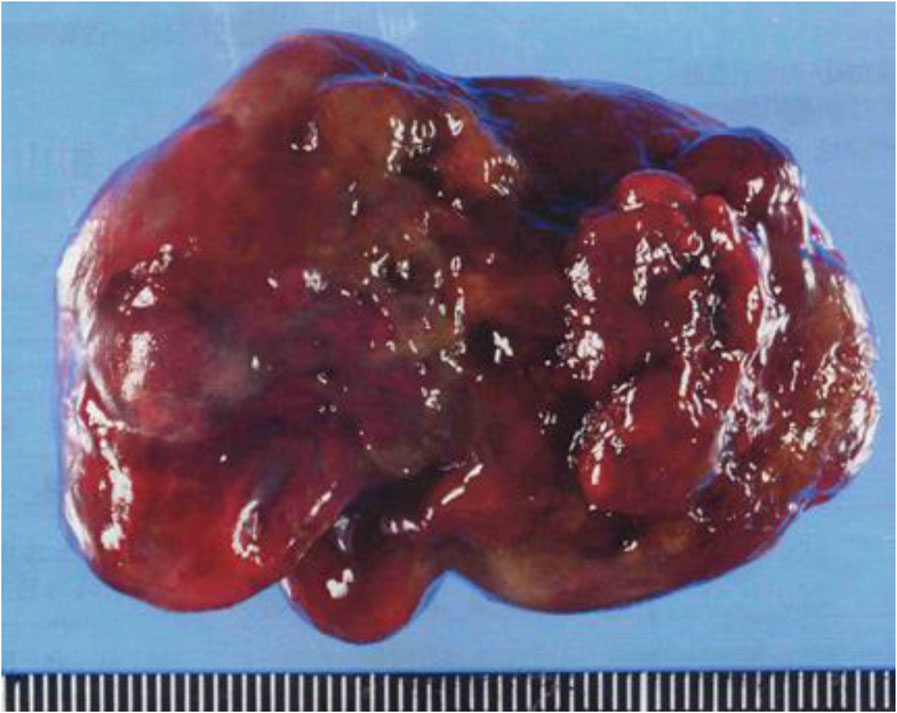
The resected specimen of the left atrial myxoma showed a multilobular structure

**Fig. 2:**
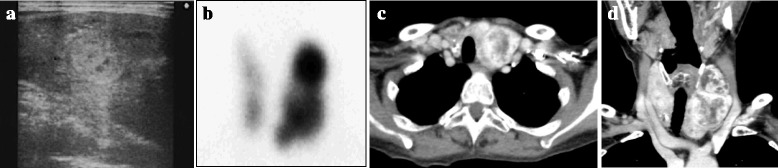
Imaging findings of the thyroid gland. Ultrasonography demonstrated multiple nodules in the thyroid gland (**a**). The thyroid nodules showed an increased uptake of ^99m^Tc on thyroid scintigraphy (**b**). The axial (**c**) and coronal view (**d**) of the thyroid gland on contrast-enhanced computed tomography showed heterogeneously enhanced multiple tumors with microcalcifications

She had been operated on 10 years earlier to extirpate a left atrial myxoma (Fig. 1). Multiple thyroid nodules were observed at that time. Thyroid scintigraphy revealed an increased focal uptake of ^99m^Tc in the same lesions in the left lobe, being consistent with toxic multinodular goiter. These corresponded to the criteria of CNC, and she was diagnosed with CNC. Genetic studies found no mutation in the PRKAR1A gene. From that time on, she received annual brain MRI, abdominal CT, and thyroid US examinations. The thyroid nodules on thyroid US gradually increased in number and size. Cervical CT showed heterogeneously enhanced masses with microcalcifications in the thyroid gland, but no apparent lymph node swelling was found (Fig. 2). Aspiration cytology revealed the nodules to be class III, but the possibility of malignant tumors could not be ruled out. We decided to perform a partial thyroidectomy with sampling of regional lymph nodes.

**Fig. 3:**
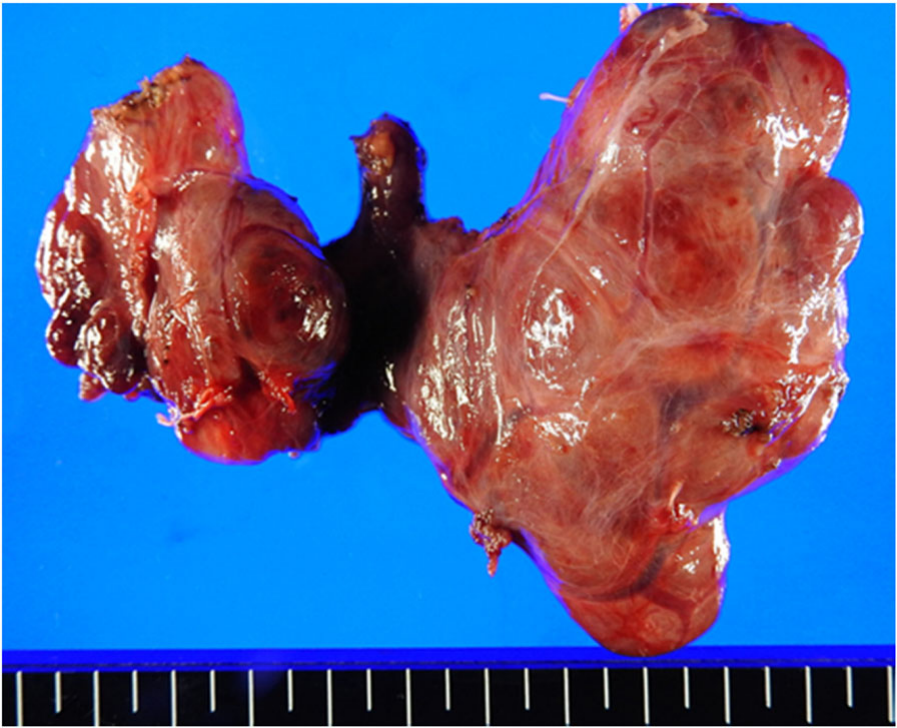
The resected specimen of the thyroid gland. The surface of the thyroid gland was rugged and slightly hard. It included multiple nodules with a maximal size of 4.0cm in diameter

**Fig. 4:**
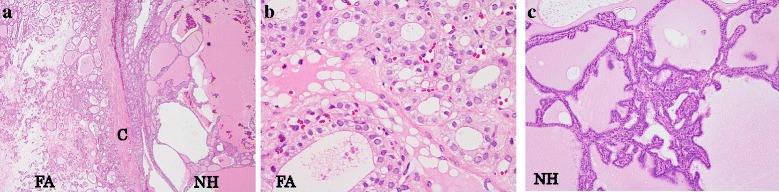
Histology of the thyroid gland. **a** Multiple follicular adenomas with a thick fibrous capsule were found in the bilateral lobes. Nodular hyperplasia was scattered in the background of the follicular adenomas (H & E stain x2 objective, FA: follicular adenoma, NH: nodular hyperplasia, C: capsule). **b** Most follicular adenomas had the characteristics of the oxyphilic cell variant (H & E stain x40 objective). **c** Nodular hyperplasia had colloid-filled follicles of variable size. It also had collections of small follicles projecting into the lumen of large actively secreting follicles (Sanderson pollsters) (H & E stain; x10 objective ). There was no evidence of malignancy and lymph nodes were normal

The resected specimen is shown in Fig. 3. The surface was rugged and slightly hard. It included multiple nodules with a maximum size of 4.0 cm. Microscopically, multiple follicular adenomas were present in the bilateral lobes. Most of them had the characteristics of the oxyphilic cell variant. Nodular hyperplasia was scattered in the background of the tumor (Fig. 4). There was no malignancy, and lymph nodes were normal.

The patient was discharged on the 7th day after surgery following an uneventful postoperative course and has been well ever since.

### Discussion

The characteristic symptoms of CNC are myxomatous masses (cardiac, cutaneous, and breast), pigmented lesions (lentigines and blue nevi), and endocrine overactivity. Diagnostic criteria for CNC were proposed by Stratakis CA in 2001 (Table 1) [3]. In Japan, since the first report of Akama, only 33 cases of CNC have been reported to date.

The major worldwide reviews of CNC [3–5] and the 33 Japanese CNC cases [6–20] are summarized in Tables 2 and 3, respectively. Spotty skin pigmentation, cardiac myxomas, cutaneous myxomas, primary pigmented nodular adrenocortical disease (PPNAD), and pituitary adenoma were the common clinical manifestations of CNC globally as well as in Japan. Meanwhile, osteochondromyxoma, psammomatous melanotic schwannoma, and blue nevi were rare in Japan. The discrepancy may reflect genetic differences among racial groups. It is therefore important to consider these regional manifestations in making a differential diagnosis of CNC. Most CNC patients in Japan initially visited a dermatologist or endocrine physician, ultimately leading to a later CNC diagnosis. This trend reflects the chief complaints of these CNC patients, which were Cushing sign (33%), palpation of the subcutaneous or breast tumor (27%), and pigment deposition of the skin (24%). It is thus difficult to make a definitive diagnosis of CNC in a patient’s first visit, and we should keep in mind the potential existence of CNC when making a differential diagnosis.

**Table 1:** Diagnostic criteria for CNC by Stratakis CA in 2001 [3]

Major Criteria	
1.	Spotty skin pigmentation with a typical distribution (lips, conjunctiva and inner or outer canthi, vaginal and penile mucosa)
2.	Myxoma (cutaneous and mucosal)
3.	Cardiac myxoma
4.	Breast myxomatosis or fat-suppressed magnetic resonance imaging findings suggestive of this diagnosis
5.	PPNAD or paradoxical positive response of urinary glucocorticosteroids to dexamethasone administration during Liddle’s test
6.	Acromegaly due to GH-producing adenoma
7.	LCCSCT or characteristic calcification on testicular ultrasonography
8.	Thyroid carcinoma or multiple, hypoechoic nodules on thyroid ultrasonography, in a young patient
9.	Psammomatous melanotic schwannoma
10.	Blue nevus, epithelioid blue nevus (multiple)
11.	Breast ductal adenoma (multiple)
12.	Osteochondromyxoma
Supplemental criteria	
1.	Affected first-degree relative
2.	Inactivating mutation of the PRKAR1A gene

*PPNAD* primary pigmented nodular adrenocortical disease; *GH* growth hormone; *LCCSCT* large-cell calcifying Sertoli cell tumor

**Table 2:** Clinical manifestations of Carney Complex in previous reports

	Carney [4])/1985 n=40	Stratakis [3]/2001 n=338	Correa [5]/2015 n=750
Gender			
Male	40	43	37
Female	60	57	63
Family history	25	70	70
Clinical manifestations			
Spotty skin pigmentation	65	77	70-80
Cardiac myxoma	72	53	20-40
Cutaneous myxoma	45	33	30-50
Primary pigmented nodular adrenocortical disease (PPNAD)	45	26	25-60
Large-cell calcifying Sertoli cell tumor or steroid-type tumor or both	56	33	41
Mammary myxomatosis or fibroadenoma	42	3	20
Pituitary adenoma (acromegaly)	10	10	75
Thyroid tumors	0	5	75
Osteochondromyxoma	0	2	rare
Psammomatous melanotic schwannoma	5	10	10
Blue nevus	0	0	75
Mutation of PRKAR1A gene	NA	41	70-80

The table gives the percentage of CNC patients

*NA* not available

**Table 3:** Clinical features of 33 Carney Complex patients reported in Japan

Clinical features	No. of patients	%
Age	mean, 30.3 years (range; 3-73 years)	
with PRKAR1A mutation	mean, 26.6±20.2 (n=7)	
without PRKAR1A mutation	mean, 64.3±11.7 (n=3)	
not available	mean, 27.0±15.5 (n=23)	
Gender		
Male	7	21
Female	26	79
Family history		
yes	13	39
no	20	61
Chief complaints		
subcutaneous or beast tumor	9	27
pigment deposition of the skin	8	24
Cushing sign	11	33
others	1	3
no symptom	4	12
Clinical manifestations		
spotty skin pigmentation	16	49
cardiac myxoma	14	42
cutaneous myxoma	13	39
primary pigmented nodular adrenocortical disease (PPNAD)	17	52
large-cell calcifying Sertoli cell tumor or steroid-type tumor or both	1	3
mammary myxomatosis or fibroadenoma	7	21
pituitary adenoma (acromegaly)	13	39
thyroid tumors	5	15
osteochondromyxoma	0	0
psammomatous melanotic schwannoma	0	0
blue nevus	0	0
Operation history		
none	6	18
once	15	46
twice	6	18
more than three times	6	18

PRKAR1A genetic mutation helps with CNC diagnosis, and it is included in the supplementary diagnostic criteria. PRKAR1A was identified in 2000 as a causative gene of CNC type 1, but not of CNC type 2 [1]. PRKAR1A gene encodes the regulatory subunit type 1-α of protein kinase A (PKA, cAMP-dependent protein kinase). Activated PKA promotes phosphorylation of CREB (cAMP response element-binding protein) that relates to the copying of cells, metabolism, and cell cycle progress. PRKAR1A inhibits these PKA pathways, and the PRKAR1A gene is a tumor suppressor gene. Only 10 Japanese CNC cases, including our case, have received the PRKAR1A genetic test, and most of them were recent, between 2013 and 2017, indicating that the genetic test has not been widespread in clinical practice. Seven of the 10 CNC cases which included the PRKAR1A genetic test showed PRKAR1A genetic mutation (Table 3), and these patients were younger than those without the mutation (mutation group 26.6 ± 20.2 years old, no mutation group 64.3 ± 11.7 years old, *p* = 0.0339). Our patient with no PRKAR1A mutation was diagnosed with CNC at the age of 59 years old.

In CNC patients, primary tumors are present synchronously and/or metachronously in multiple organs, including cardiac myxomas, pituitary tumors, breast tumors, adrenocortical tumors, and thyroid tumors, and they require repeated surgeries over several years. Six of the 33 (18%) Japanese CNC patients underwent surgery more than three times. The CNC patients should thus be monitored closely for clinical manifestations of the disease and be aware of necessity for polysurgery to improve prognoses.

Worldwide, up to 60% of CNC patients had thyroid nodules; nonspecific cystic disease was present in 75%, follicular adenomas in 25%, and papillary or follicular carcinomas in up to 10% of the cases [5]. Whereas in Japan, only 5 of 33 (15%) CNC cases showed thyroid nodules, including carcinoma [8–11], as shown in Table 4. All patients were female, and the average age at detection of thyroid lesions was 56.2 years old. The CNC patients with PRKAR1A genetic mutation were more susceptible to the occurrence of thyroid tumors, and about two thirds of these cases showed thyroid disorder in infancy or adolescence [2, 5]. The age at onset of thyroid cancer in two Japanese CNC patients was 72 and 73 years old, in whom PRKAR1A mutation was negative in one and not available in the other. However, the thyroid tumors were often detected by chance during a general medical examination because most patients were not conscious of any nodule or swelling of the thyroid gland, except in our case.

**Table 4:** Clinical characteristics of the 5 Carney Complex patients with thyroid lesion reported in Japan

No.	Author/Year/Ref. no.	Age(y)/Sex	Family history	Carney complex manifestations	PRKAR1A mutation
1	Ando et al./2015/ [9]	72/F	no	Thyroid tumor, lentigines, pituitary adenoma (TSH-producing tumor)	NA
2	Okamoto et al./2017/ [8]	73/F	no	Thyroid tumor, lentigines, pituitary adenoma (TSH-producing tumor)	none
3	Kako et al./1999/ [11]	47/F	no	Thyroid tumor, lentigines, cardiac myxoma, PPNAD	NA
4	Yamashita et al./2015/ [10]	20/F	no	Thyroid tumor, breast myxoma, cardiac myxoma, PPNAD, acromegaly	NA
5	Our case/2017	69/F	no	Thyroid tumor, cardiac myxoma	none

*TSH* thyroid stimulating hormone; *PPNAD* primary pigmented nodular adrenocortical disease; *NA* not available

The evaluation and treatment of thyroid lesions in the 5 Japanese CNC patients are summarized in Table 5. Although CNC patients usually show thyroid function within the normal range, two Japanese cases with thyroid lesions, including our patient, had a high TSH level (Table 5). Our patient had taken thiamazole for an extended period of time for hyperthyroidism; the negative feedback mechanism of which might have been involved in the high TSH level. The other case had a TSH-producing pituitary adenoma. Although TSH-producing pituitary adenomas account for only 0.2~1.0% of total pituitary adenomas, 3 of the 33 (9.1%) Japanese CNC patients had TSH-producing pituitary adenomas. The correlation between TSH value and occurrence of thyroid cancer has not been demonstrated in CNC patients, but the adenylate cyclase-PKA pathway located downstream of the TSH receptor may be involved in the development of thyroid cancer. Therefore, the monitoring of TSH levels may be important to allow for an early detection of thyroid cancer in CNC patients. During the long follow-up, any increase in the number, size, and calcification of the thyroid lesions could be a sign of malignancy. Our patient underwent surgery because the thyroid nodules gradually increased in number and size on follow-up thyroid US examinations. Four of 5 Japanese CNC patients with thyroid lesion underwent a partial thyroidectomy, 2 with cancer and 2 with adenoma, histologically. It is possible that thyroid adenomas in CNC could increase and grow over a long period and have the potential for malignant transformation based on an adenoma-carcinoma sequence. Cardiovascular disease is the leading cause of death for CNC patients [3]. Thyroid-related death has rarely been reported, and thyroid cancer in CNC has a good prognosis due to their well-differentiated nature.

**Table 5:** Evaluation and treatment for thyroid lesion in the 5 Carney Complex patients reported in Japan

No.	Author	Thyroid exam.	Free T3 (pg/mL)	Free T4 (ng/dL)	TSH (μIU/mL)	Ultrasonography findings	Treatment	Pathology
1	Ando [9]	Within normal	2	1.16	9.9	Multiple hypoechoic lesions, microcalcification	Partial thyroidectomy	Thyroid cancer
2	Okamoto [8]	Within normal	5.47	2.27	3.22	Multiple hypoechoic lesions, microcalcification	Partial thyroidectomy	Thyroid cancer (papillary)
3	Kako [11]	Within normal	NA	NA	NA	NA	Partial thyroidectomy	Follicular adenoma
4	Yamashita [10]	Within normal	NA	NA	NA	Single hypoechoic lesion	follow-up	NA
5	Our case	Nodule	3.05	1.05	4.56	Multiple hypoechoic lesions, microcalcification	Partial thyroidectomy	Follicular adenoma

The normal value: Free T3, 2.51-4.12pg/mL ; Free T4, 0.88-1.50ng/dl ; TSH, 0.464-3.725μIU/ml

*NA* not available

In summary, the characteristics of thyroid lesions in CNC are as follows: (1) the thyroid lesions are present without major symptoms and are usually accompanied by other neoplastic syndromes in various organs, (2) adenomas are the predominant pathology of thyroid lesions, and they tend to increase in size and number during a long follow-up, (3) the age of onset of thyroid lesions is younger in cases with PRKAR1A genetic mutation, and (4) thyroid cancer develops in 6% of CNC patients, but they are well-differentiated cancers, i.e., papillary or follicular cancer, with favorable prognoses.

## Conclusions

Data on thyroid cancers and non-cancerous thyroid tumors associated with CNC has become increasingly available due to the accumulation of cases and advances in molecular genetic studies in recent years. Thyroid carcinomas can develop in the background of a variety of follicular thyroid lesions in CNC patients despite biochemical euthyroid status and no palpable mass. Long-term follow-up examinations of the whole body, including the thyroid gland, are essential in CNC patients.

## Abbreviations

CNC: Carney complex
CREB: cAMP response element-binding protein
CT: Computed tomography
MRI: Magnetic resonance imaging
PKA: Protein kinase A
PPNAD: Primary pigmented nodular adrenocortical disease
PRKAR1A: Protein kinase A regulatory subunit 1-α
TSH: Thyroid-stimulating hormone
US: Ultrasonography
